# Effect of intraoperative haemoadsorption therapy on cardiac surgery for active infective endocarditis with confirmed *Staphylococcus aureus* bacteraemia

**DOI:** 10.1093/icvts/ivad010

**Published:** 2023-01-27

**Authors:** Zaki Haidari, Spela Leiler, Hazem Mamdooh, Matthias Fittkau, Kristina Boss, Bartosz Tyczynski, Matthias Thielmann, Erik Bagaev, Mohamed El Gabry, Daniel Wendt, Andreas Kribben, Thomas Bertsch, Arjang Ruhparwar, Theodor Fischlein, Jurij Matija Kalisnik

**Affiliations:** Department of Thoracic and Cardiovascular Surgery, West German Heart and Vascular Center Essen, University Hospital Essen, Essen, Germany; Department of Cardiac Surgery, Klinikum Nürnberg, Paracelsus Medical University, Nuremberg, Germany; Department of Cardiac Surgery, Klinikum Nürnberg, Paracelsus Medical University, Nuremberg, Germany; Department of Cardiac Surgery, Klinikum Nürnberg, Paracelsus Medical University, Nuremberg, Germany; Department of Nephrology, University Hospital Essen, Essen, Germany; Department of Nephrology, University Hospital Essen, Essen, Germany; Department of Thoracic and Cardiovascular Surgery, West German Heart and Vascular Center Essen, University Hospital Essen, Essen, Germany; Department of Cardiac Surgery, Klinikum Nürnberg, Paracelsus Medical University, Nuremberg, Germany; Department of Thoracic and Cardiovascular Surgery, West German Heart and Vascular Center Essen, University Hospital Essen, Essen, Germany; Department of Thoracic and Cardiovascular Surgery, West German Heart and Vascular Center Essen, University Hospital Essen, Essen, Germany; Cytosorbents Inc., Princeton, NJ, USA; Department of Nephrology, University Hospital Essen, Essen, Germany; Institute of Clinical Chemistry, Laboratory Medicine and Transfusion Medicine, Paracelsus Medical University, Nuremberg, Germany; Department of Thoracic and Cardiovascular Surgery, West German Heart and Vascular Center Essen, University Hospital Essen, Essen, Germany; Department of Cardiac Surgery, Klinikum Nürnberg, Paracelsus Medical University, Nuremberg, Germany; Department of Cardiac Surgery, Klinikum Nürnberg, Paracelsus Medical University, Nuremberg, Germany

**Keywords:** Infective endocarditis, *Staphylococcus aureus*, Cardiac surgery, Haemoadsorption, Sepsis

## Abstract

**OBJECTIVES:**

Sepsis caused by infective endocarditis (IE), due to *Staphylococcus aureus*, is associated with significant morbidity and mortality. Blood purification using haemoadsorption (HA) may attenuate the inflammatory response. We investigated the effect of intraoperative HA on postoperative outcomes in *S. aureus* IE.

**METHODS:**

Patients with confirmed *S. aureus* IE undergoing cardiac surgery were included in a dual-centre study between January 2015 and March 2022. Patients treated with intraoperative HA (HA group) were compared to patients not treated with HA (control group). The primary outcome was vasoactive-inotropic score within the first 72 h postoperatively and secondary outcomes were sepsis-related mortality (SEPSIS-3 definition) and overall mortality at 30 and 90 days.

**RESULTS:**

No differences in baseline characteristics were observed between groups (haemoadsorption group, *n* = 75, control group, *n* = 55). Significantly decreased vasoactive-inotropic score was observed in the haemoadsorption group at all time points [6 h: 6.0 (0–17) vs 17 (3–47), *P* = 0.0014; 12 h: 2 (0–8.3) vs 5.9 (0–37), *P* = 0.0138; 24 h: 0 (0–5) vs 4.9 (0–23), *P* = 0.0064; 48 h: 0 (0–2.1) vs 0.1 (0–13), *P* = 0.0192; 72 h: 0 (0) vs 0 (0–5), *P* = 0.0014]. Importantly, sepsis-related mortality (8.0% vs 22.8%, *P* = 0.02) and 30-day (17.3% vs 32.7%, *P* = 0.03) and 90-day overall mortality (21.3% vs 40%, *P* = 0.03) were also significantly lower with haemoadsorption.

**CONCLUSIONS:**

Intraoperative HA during cardiac surgery for *S. aureus* IE was associated with significantly lower postoperative vasopressor and inotropic requirements and resulted in lower sepsis-related and overall 30- and 90-day mortality. In this high-risk population, improved postoperative haemodynamic stabilization by intraoperative HA appears to improve survival and should be further tested in future randomized trials.

**Figure:**
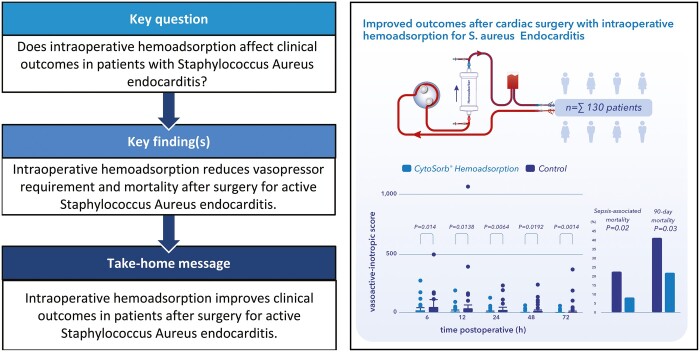

## INTRODUCTION

Postoperative sepsis followed by organ failure is an important cause of morbidity and mortality in patients undergoing cardiac surgery for infective endocarditis (IE) [1]. *Staphylococcus aureus* (*S. aureus*) is the leading cause of IE, and the significantly increased mortality rate has remained high despite increasingly effective diagnostic and therapeutic procedures [2, 3]. The poorer outcomes observed in patients undergoing cardiac surgery with confirmed culture-positive IE and *S. aureus*, in particular, are likely due to the frequent occurrence of postoperative sepsis resulting from the combination of a cytokine driven systemic inflammatory response (SIR) and underlying systemic infection [4–8]. Blood purification using haemoadsorption (HA) therapy is increasingly used in high-risk cardiac surgery cases, including acute IE, to modulate SIR and improve postoperative outcomes [9]. Blood purification, in addition to removal of cytokines, HA is able to remove S. aureus toxin and haemolysin from blood [10], what may make it a particularly attractive intervention for patients with confirmed *S. aureus* IE.

Sepsis and septic shock are still not completely understood, but it is well-established that the release of both pro- and anti-inflammatory mediators play a central role. The SIR is orchestrated in large part by the high levels of circulating cytokines, a phenomenon termed ‘cytokine storm’ in the most severe cases. Blood purification by HA is capable to remove excessive pro-inflammatory and potentially toxic cytokines (such as IL-6, IL-10, TNFα or MCP-1) [11].

Therefore, the aim of the present study was to evaluate the clinical effects of intraoperative HA in confirmed *S. aureus* IE patients undergoing cardiac surgery.

## METHODS

### Patients

Eligible participants for the study included consecutive patients operated on for definite IE according to the modified Duke criteria (2 major or 5 minor or 1 major + 3 minor criteria) at the 2 participating centres (West German Heart & Vascular Center, Dept. of Thoracic- and Cardiovascular Surgery, Essen, Germany, and Department of Cardiac Surgery, Klinikum Nürnberg, Nuremberg, Germany) between January 2015 and March 2022. The present analysis was a retrospective evaluation of prospectively collected data. Only patients with HA being intraoperatively applied were included in the present analysis. The final study population was then limited to patients with blood culture-confirmed systemic *S. aureus* [both, methicillin-susceptible *S. aureus* (MSSA) and methicillin-resistant S. aureus (MRSA)]. Finally, cardiac device-related IE requiring simple retraction without cardiopulmonary bypass (CPB) or isolated tricuspid IE was excluded. EuroSCORE II was calculated using the online calculator (http://www.euroscore.org/calc.html). The study was performed in accordance with the Declaration of Helsinki and the International Conference on Harmonization Good Clinical Practice guidelines and was reviewed and approved by the institutional ethics committee and Institutional Review Board (19-8743-BO) in Essen and by the Institutional Study Centre (SZ_W_134.21-I-6) and Institutional Review Board (IRB-2021-031) in Nuremberg. An informed consent was waived due to anonymity.

### Outcome measures

The primary outcome was haemodynamic stability during the postoperative course over the first 72 h, defined by the vasoactive-inotropic score (VIS). Secondary outcomes included the incidence of sepsis-related mortality (according to SEPSIS-3 guidelines) [12], 30- and 90-day overall mortality and the Sequential Organ Failure Assessment (SOFA) score [13]. Sepsis was defined as life-threatening organ dysfunction due to a dysregulated host response to infection; septic shock was characterised by profound circulatory, cellular, and metabolic abnormalities with vasopressor requirement to maintain a mean arterial pressure of 65 mmHg or greater and serum lactate level greater than 2 mmol/l (>18 mg/dl) in the absence of hypovolaemia and sepsis-related death was mortality related to severe sepsis or septic shock. The total postoperative SOFA was assessed preoperatively up to the 7th postoperative day and was calculated on a scale ranging from 0 to 6 for each organ system, resulting in a total score from 0 to 24 [13].

### Definition of vasoactive-inotropic score calculation

The VIS was calculated using the following formula: VIS = dopamine dose (μg/kgBW/min) + dobutamine dose (μg/kgBW/min) + 100 × epinephrine dose (μg/kgBW/min) + 100 × norepinephrine dose (μg/kgBW/min) + 10 × milrinone dose (μg/kgBW/min) + 10 000 × vasopressin dose (IU/kgBW/min) as a dimensionless unit.

### Operative techniques

Cardiac surgery was performed under general anaesthesia and endotracheal intubation. Preoperative transoesophageal echocardiography was performed to evaluate cardiac and valvular function. Standard aortic and caval cannulation techniques were applied. Intraoperative HA was routinely used in all consecutive patients since 2018. In the cases selected for intraoperative HA, a HA device, Cytosorb^®^ (Cytosorbents, Princeton, NJ, USA), was installed in a parallel circuit of the CPB machine during the surgical procedure at a flow ranging between 100 and 700 ml. Concomitant pathologies, although not related to IE, were treated simultaneously (such as significant tricuspid/mitral valve regurgitation, coronary artery disease or patent foramen ovale).

### Haemoadsorption therapy

**Figure 1:**
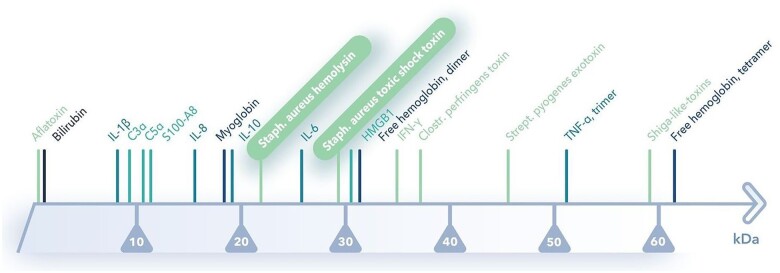
(**A**) Spectrum of adsorption and (**B**) haemoadsorption device (CytoSorbents, Princeton, NJ, USA).

HA therapy with CytoSorb^®^ is based on the extracorporeal blood purification that reduces excessive levels of inflammatory mediators with the aim of modulating the exaggerated immune response by reducing elevated levels of circulating cytokines and mitigating their detrimental downstream effects. In addition to removing cytokines, HA is able to remove *S. aureus* toxin and haemolysin from blood [10]. CytoSorb^®^ can be easily integrated into various extracorporeal circuits, such as continuous renal replacement therapy, CPB and extracorporeal membrane oxygenation. The device is filled with highly biocompatible, porous polymer beads covered with a divinylbenzen coating. Each polymer bead is between 300 and 800 μm in size and has pores and channels, giving it a large (40 000 m^2^) effective surface area for binding hydrophobic small- and middle-size molecules. Figure 1 shows the spectrum of adsorption and the device.

### Statistical analysis

Data were analysed using SPSS software version 25 (SPSS Inc., Chicago, IL, USA). Continuous variables were expressed as mean standard deviation (SD) and the SOFA and VIS were presented as median and interquartile range, respectively, and compared using Student’s *t*-test or the Wilcoxon rank-sum test. Categorical data were expressed as number of patients and frequencies and compared using the chi-squared test. A *P*-value of <0.05 was considered statistically significant.

## RESULTS

### Baseline characteristics

From January 2015 to March 2022, a total of 130 patients underwent cardiac surgery with CPB for IE with confirmed *S. aureus* infection. Seventy-five patients received intraoperative HA (HA group, *n* = 75), while 55 patients operated on without HA served as controls (control group, *n* = 55). Preoperative baseline characteristics of the patients and types of valves affected are displayed in Table 1. The mean EuroSCORE II for both groups was 11.9% and 12.0%, indicating a high-risk surgical population. The mean time between definitive diagnosis of IE and surgery was 12.6 (SD: 13.7) days in the HA group compared with 10.3 (SD: 9.6) in controls (*P* = 0.27). The mean duration of preoperative antibiotic treatment was 11.5 (SD: 10.2) days versus 9.7 (SD: 8.3) days (*P* = 0.47) in the HA and control groups, respectively. Before induction of anaesthesia, 9 patients in the HA group and 8 in the control group were intubated and about 12% of the patients in both groups needed vasopressor or inotropic therapy. There were no statistical differences in terms of baseline demographics, comorbidities or preoperative haemodynamic and pulmonary status. Overall, 82 valves in the HA group and 68 valves in the control group were affected. Combined procedures were equally represented in both groups. In all patients, MSSA or MRSA was confirmed.

**Table 1:** Baseline characteristics

Variable	HA group (*n* = 75)	Control group (*n* = 55)	*P*-Value
Demographics			
Age (years)	60.7 ± 16.5	60.8 ± 13.5	0.99
Gender, male	38 (50.1)	32 (58.2)	0.47
Systemic hypertension	47 (62.7)	35 (63.6)	1.00
Coronary artery disease	25 (33.3)	13 (23.6)	0.24
COPD	12 (16.0)	10 (18.2)	0.81
Pulmonary hypertension	4 (5.3)	3 (5.5)	1.00
Dialysis dependent	6 (8.0)	5 (9.1)	1.00
Liver cirrhosis	3 (4.0)	2 (3.6)	1.00
Peripheral vascular disease	6 (8.0)	9 (16.3)	0.17
Atrial fibrillation	26 (34.7)	12 (21.8)	0.12
IV drug abuse	10 (13.3)	12 (21.8)	0.24
Re-operation	19 (25.2)	15 (27.2)	0.84
LV ejection fraction	52.0 ± 10.7	55.2 ± 6.7	0.32
History of stroke	28 (37.3)	18 (32.7)	0.71
Fever (≥38.5°C)	57 (76.0)	45 (81.8)	0.51
EuroSCORE II (%)	11.9 ± 15.2	12.0 ± 11.5	0.49
SOFA score, preoperative	1 (0–3)	2 (0–5)	0.38
Leucocytes (nl)	12.3 ± 5.7	10.0 ± 4.6	0.005
CRP (mg/dl)	10.3 ± 8.3	8.7 ± 7.4	0.13
Clinical status			
Intubated	9 (12.0)	8 (14.5)	0.79
Vasopressor need	9 (12.0)	7 (12.7)	1.000
*Staphylococcus* species			
MSSA	69 (92.0)	52 (94.5)	0.73
MRSA	6 (8.0)	3 (5.5)	0.73
Affected valves			
MV	28 (37.3)	25 (45.5)	0.37
AV	27 (36.0)	23 (41.8)	0.58
MVP	6 (8.0)	2 (3.6)	0.46
AVP	18 (24.0)	10 (18.2)	0.52
TV	3 (4.0)	8 (14.5)	0.05
Multiple valves	7 (13.3)	8 (14.5)	0.41
Total number of valves	82	68	

Data are presented as mean ± SD or number (%). SOFA score is presented as median and IQR.

AV: aortic valve; AVP: aortic valve prosthesis; COPD: chronic obstructive pulmonary disease; EuroSCORE: European System for Cardiac Operative Risk Evaluation; HA: haemoadsorption; IQR: interquartile range; IV: intravenous; MRSA: methicillin-resistant *Staphylococcus aureus*; MSSA: methicillin-susceptible *Staphylococcus aureus*; MV: mitral valve; MVP: mitral valve prosthesis; SD: standard deviation; SOFA: Sequential Organ Failure Assessment; TV: tricuspid valve; LV: left ventricular; CRP: C-reactive protein.

### Operative characteristics

Table 2 represents operative characteristics of the 2 groups. Indications for surgery were mainly: heart failure, large mobile vegetations, uncontrolled infection or a severe valve dysfunction (mainly regurgitation). Prosthetic valve IE was present in 24 patients from the HA group versus 12 patients in the control group. Concomitant coronary artery bypass grafting was performed in 9 patients in the HA group and 6 patients in the control group. CPB and aortic cross-clamp times were comparable between the 2 groups.

**Table 2:** Operative characteristics

Variable	HA group (*n* = 75)	Control group (*n* = 55)	*P*-Value
MV repair	19 (25.3)	10 (18.2)	0.39
MV replacement	17 (22.7)	18 (32.7)	0.23
AV replacement	47 (62.7)	33 (60.0)	0.85
TV repair	6 (8.0)	8 (14.5)	0.26
Prosthetic valve endocarditis	24 (32.0)	12 (21.8)	0.24
Concomitant CABG	9 (12.0)	6 (10.9)	1.00
Cardiopulmonary bypass time (min)	133.2 ± 72.8	142.4 ± 85.6	0.51
Aortic cross-clamp time (min)	89.8 ± 46.6	91.4 ± 48.3	0.84

Data are presented as mean ± SD or number (%).

AV: aortic valve; CABG: coronary artery bypass grafting; HA: haemoadsorption; MV: mitral valve; SD: standard deviation; TV: tricuspid valve.

### Outcomes

**Figure 2:**
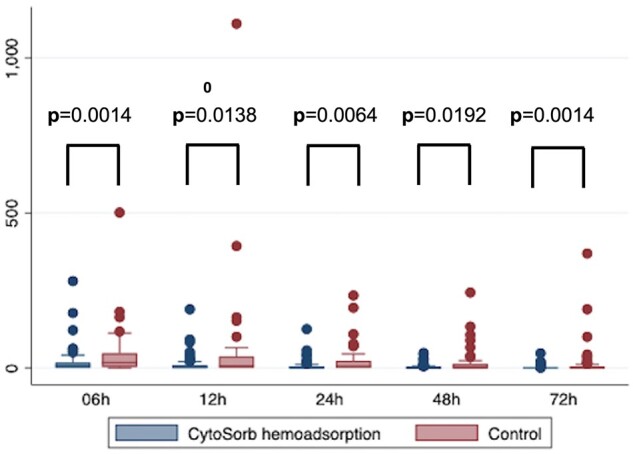
Box plot illustration of the vasoactive-inotropic score during the postoperative course.

**Figure 3:**
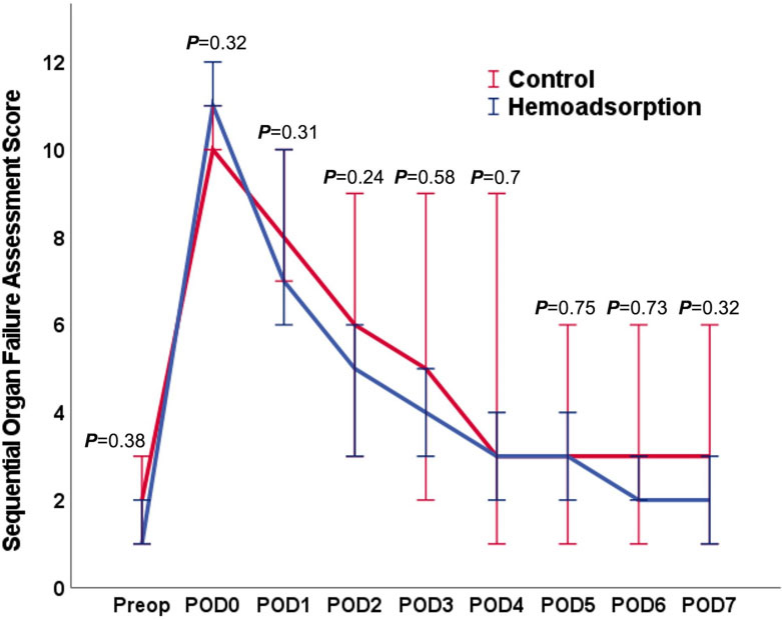
SOFA score during the postoperative course. SOFA: Sequential Organ Failure Assessment.

The primary outcome was the vasopressor requirement for postoperative haemodynamic stabilization within the first 72 h after surgery. Cumulative vasopressor requirements were assessed using the standardized VIS and are summarized in Fig. 2 and Table 3. For each timepoint (6, 12, 24, 48 and 72 h), the HA group had significantly lower VIS compared with controls (*P* < 0.05 for all timepoints). The relative reductions in VIS also remained consistent over 72 h ranging between 59% and 82%. Sepsis-related death occurred in 6 patients in the HA group and in 12 patients in the control group (*P* = 0.02). Overall mortality was 17.3% in the HA group and 32.7% in the control group at 30 days (*P* = 0.03) and 21.3% vs 40.0% at 90 days (*P* = 0.03). This corresponds to a number needed to treat of 5 patients to save 1 life (at 90 days) and a relative mortality risk reduction of 46.8% compared to standard therapy. All secondary outcomes are summarized in Table 4. We did not observe any significant differences between the 2 groups in postoperative SOFA scores with results depicted in Fig. 3 and Table 4.

**Table 3:** Primary outcome vasoactive-inotropic score

Vasoactive-inotropic score	HA group (*n* = 75)	Control group (*n* = 55)	*P*-Value
6 h	6 (0–17)	17 (3–47)	0.0014
12 h	2 (0–8.3)	5.9 (0–37)	0.0138
24 h	0 (0–5)	4.9 (0–23)	0.0064
48 h	0 (0–2.1)	0.1 (0–13)	0.0192
72 h	0 (0)	0 (0–5)	0.0014

Vasoactive-inotropic score is presented as median and IQR.

IQR: interquartile range.

**Table 4:** Secondary and postoperative outcomes

Variable	HA group (*n* = 75)	Control group (*n* = 55)	*P*-Value
Mortality			
Sepsis-associated mortality	6 (8.0)	12 (21.8)	0.02
30-Day mortality	13 (17.3)	18 (32.7)	0.03
90-Day mortality	16 (21.3)	22 (40.0)	0.03
Outcomes			
Postoperative IABP/ECMO	4 (5.3)	4 (7.2)	1.00
New stroke	5 (6.7)	3 (5.5)	1.00
New dialysis	16 (21.3)	22 (40.0)	0.03
Revision for bleeding	7 (9.3)	12 (21.8)	0.07
Reintubation	10 (13.3)	6 (10.9)	0.79
ICU stay (days)	9.8 ± 15.5	8.3 ± 10.3	0.27
Hospital stay (days)	21.8 ± 18.9	24.6 ± 19.6	0.20
Leucocytes (nl)	17.7 ± 8.6	23.6 ± 9.1	0.09
CRP (mg/dl)	6.6 ± 5.0	9.9 ± 8.0	0.005
SOFA score, preoperative	1 (0–3)	2 (0–5)	0.38
SOFA score, day 0	11 (9–13)	10 (9–12)	0.32
SOFA score, day 1	7 (4–11)	9 (4–12)	0.31
SOFA score, day 2	5 (2–9)	6 (2–11)	0.24
SOFA score, day 3	4 (2–7)	5 (1–10)	0.58
SOFA score, day 4	3 (1–6)	4 (0–10)	0.70
SOFA score, day 5	3 (1–6)	3 (0–9)	0.75
SOFA score, day 6	2 (1–5)	3 (0–9)	0.73
SOFA score, day 7	2 (1–5)	2 (0–9)	0.32

Data are presented as mean ± SD or number (%). SOFA score is presented as median and IQR.

ECMO: extracorporeal membrane oxygenation; HA: haemoadsorption; IABP: intra-aortic balloon pump; ICU: intensive care unit; IQR: interquartile range; SD: standard deviation; SOFA: Sequential Organ Failure Assessment.

Postoperative parameters showed that mechanical circulatory support with extracorporeal membrane oxygenation was necessary in 4 patients, 2 in each respective group. Intra-aortic balloon pump support was applied in 4 patients, again 2 from each group. A new stroke occurred in 3 patients from the controls and 5 patients from the HA group (*P* = ns). New, postoperative renal failure requiring haemodialysis developed in 38 patients, 16 in the HA group and 22 in the control group (*P* = 0.03). Re-operation for bleeding was required in 7 patients in the HA group and 12 patients in the control group (*P* = 0.07). Respiratory failure requiring reintubation occurred in 16 patients, 10 in the HA group and 6 in the control group, *P* = 0.79. There were no clinically relevant differences in the intensive care unit and hospital lengths of stay between the groups. All postoperative outcome parameters are shown in Table 3.

## DISCUSSION

There were 3 main observations in the current study. First, patients undergoing cardiac surgery for acute *S. aureus* IE remain at high risk for postoperative complications and continue to experience high mortality rates (28% at 90 days). Second, intraoperative HA was easy to use, safe and significantly reduced postoperative vasopressor and inotropic requirements. Third, the use of intraoperative HA was associated with significantly fewer sepsis-associated deaths and resulted in significantly lower overall mortality.

To the best of our knowledge, this study is the first to evaluate the effect of intraoperative HA exclusively in patients with IE based on *S. aureus*. Especially in patients suffering from endocarditis caused by *Staphylococcus* species, results are devasting compared to other bacteria [14]. In patients undergoing cardiac surgery for IE, postoperative sepsis still represents an important cause of mortality and morbidity [14, 15]. The virulence of *S. aureus* is mainly defined by a huge repertoire of virulence factors, among which secreted toxins play a preeminent role [14]. HA therapy with the use of CytoSorb^®^ is known to remove hydrophobic substances up to 60 kD, which also applies to the toxin of *S. aureus* as the potential underlying mechanism for postoperative sepsis and complications. Therefore, we hypothesized that removal of the *S. aureus* toxin could potentially result in improved haemodynamics in IE patients suffering from *S. aureus* endocarditis.

IE caused by *S. aureus* is associated with high rates of postoperative sepsis and increased mortality [16, 17]. A recent multicentre analysis evaluating 4917 patients showed almost double 30-day mortality in *Staphylococcus* caused IE patients [8]. Within the present study we could confirm that mortality is still high in such a high-risk cohort presenting with IE. Interestingly, we could show that sepsis-related death and 30- and 90-day mortality could be significantly reduced by intraoperative adjunctive HA in *S. aureus* IE patients. Furthermore, this difference in mortality was reinforced by the favourable haemodynamic parameters in patients receiving intraoperative HA. As shown in Fig. 2, a significant difference in the postoperative vasopressor and inotropic need (primary outcome parameter), as defined by the well-established [18] VIS, was observed. These observations are in accord with Dunser *et al.* [19] and Roberts *et al.* [20], demonstrating a close correlation between cumulative vasopressor load and increasing vasopressor dosing intensity during the first 24 h with mortality and renal failure in patients with sepsis or septic shock. We already observed a significant reduction in inotropic support in a prior evaluation in native mitral valve IE [21]. In line, excessive inotropes were independently associated with sepsis-related and in-hospital mortality also in a recent mixed cohort of native and prosthetic left-sided endocarditis regardless of causative infective agent [14].

Meanwhile, the results of the multicentre randomized-controlled REMOVE study (Cytokine Hemoadsorption During Cardiac Surgery Versus Standard Surgical Care for Infective Endocarditis) have been published [22]. The primary outcome was the difference in change in SOFA score. The REMOVE trial failed to show a reduction in the SOFA score in the postoperative course with intraoperative HA; however, a significant reduction in cytokines could be observed [22]. Moreover, no difference in mortality between the randomized groups could be observed within the REMOVE study. In contrast, a recent non-randomized analysis evaluating only high-risk patients as defined by an increased EuroSCORE II showed a significant reduction in the postoperative course of the SOFA score in endocarditis patients receiving intraoperative HA [23]. In the present analysis, the results were consistent with the REMOVE study as we were also unable to show a difference in postoperative SOFA scores with the use of intraoperative HA. It can be speculated that the SOFA score might not be the optimal scoring system, as it was introduced in the early 90s mainly to assess the incidence of organ dysfunction/failure in septic patients on the intensive care unit and has not been validated in an IE cardiosurgical setting so far. Nevertheless, it represents a well-established and objective score to group patients and calculate their individual risks, especially in the postoperative period [23]. The present analysis, with focusing only on a subgroup of IE patients with *S. aureus*, might help to select the most responsive patients for adjunctive HA.

It is well accepted that patients with IE have an increased risk of postoperative renal failure [24, 25]. In the present study, not only reduced mortality but also renal failure (requiring dialysis) was significantly less frequent in the HA group (*P* = 0.03). Furthermore, although failing to reach a statistically significant difference, patients from the HA group experienced less re-thoracotomies for bleeding (*P* = 0.07). Especially in patients presenting with IE the coagulation system is heavily affected and sometimes impaired leading to various types of coagulopathies [26].

*Staphylococcus per se* is known as a destructive, dangerous, and versatile pathogen. In our cohort, we observed no differences in the preoperative risk profile of the patients, although they were not randomized, and all patients had definitive MRSA/MSSA infection. Recently, the current recommendations for surgical treatment of IE were reviewed by Wang and Fosbol [27]. Regarding *S. aureus*, Wang and Fosbol concluded that in the prospective, multinational International Collaboration on Endocarditis registry of IE, *S. aureus* IE complicated by sepsis was not generally treated with surgery as *S. aureus* IE is associated with more frequent and severe complications. On the contrary, the majority of patients in the present study had an uncontrolled infection, severe regurgitation or fever on preoperative examination despite antibiotic pretreatment, implying liberal surgical indication as well as advanced complexity of the operated cohort.

### Limitations

Our retrospective study focused on the effects of intraoperative HA on haemodynamics and postoperative outcomes in patients undergoing cardiac surgery with *S. aureus* IE. Although this study was not a randomized controlled trial, both groups were comparable, but a selection bias cannot be completely excluded. In addition, groups were not adjusted by any possible confounder, which might also influence the results. In fact, also the time between diagnosis and treatment as well as the preoperative antibiotic treatment interval might influence the results. Beside this, it should be acknowledged that within the present work, by nature, only the SOFA scores of the survivors could be used for final evaluation. In the present analysis, we calculated the SOFA score for each postoperative day individually and it was not used as the primary outcome. Moreover, in the present analysis, we failed to present a detailed panel of postoperative cytokines. Nevertheless, to our knowledge, this is the first study to date investigating intraoperative HA in a subset of *S. aureus* IE patients. Finally, non-contemporary time bias throughout the time period of the present analysis cannot be completely ruled out and future larger trials might include *post hoc* matching tests.

## CONCLUSION

In conclusion, this study aimed to evaluate the outcome of patients with IE caused by *S. aureus*, focusing on the postoperative need for vasopressor and inotropic support. This was based on our hypothesis that the removal of *S. aureus* endotoxin could be achieved by HA, which could be the cause for postoperative vasoplegia and poor outcomes. We could show that intraoperative HA appears to attenuate the severity of postoperative sepsis, reducing not only the need for vasopressors but also 30- and 90-day mortality in patients undergoing cardiac surgery for IE caused by *S. aureus*. Future studies are essential to confirm the current results in a larger population and to investigate the mechanisms underlying these effects.

**Conflict of interest:** Prof. M. Thielmann received speake’s fee for participation at Cytosorbents Symposium during 36^th^ EACTS annual meeting, Prof. D. Wendt has been employed by Cytosorbents Inc. since January 2022.

## Data Availability

All relevant data are within the manuscript and its Supporting Information files.

## Notes

Presented at the 36th Annual Meeting of the European Association for Cardio-Thoracic Surgery, Milano, Italy, 5–8 October 2022.

## ABBREVIATIONS

CPB: Cardiopulmonary bypass
HA: Haemoadsorption
IE: Infective endocarditis
MRSA: Methicillin-resistant *Staphylococcus aureus*
MSSA: Methicillin-susceptible *Staphylococcus aureus*
REMOVE: Cytokine Hemoadsorption During Cardiac Surgery Versus Standard Surgical Care for Infective Endocarditis
SD: standard deviation
SIR: Systemic inflammatory response
SOFA: Sequential Organ Failure Assessment
VIS: Vasoactive-inotropic score
